# Long Term Survival and Continued Complete Response of Vemurafenib in a Metastatic Melanoma Patient with BRAF V600K Mutation

**DOI:** 10.1155/2016/2672671

**Published:** 2016-02-18

**Authors:** K. Sahadudheen, Md. Rafiqul Islam, M. Iddawela

**Affiliations:** ^1^Department of Oncology, Goulburn Valley Health, Graham Street, Shepparton, VIC 3630, Australia; ^2^Research Unit, Goulburn Valley Health, Shepparton, VIC 3630, Australia; ^3^RHAC, Department of Rural Health, University of Melbourne, Shepparton, VIC 3630, Australia; ^4^Department of Anatomy and Developmental Biology, Monash University, Melbourne, VIC 3800, Australia

## Abstract

*Introduction*. BRAF kinase inhibitors such as Vemurafenib have shown improvement in overall survival, progression-free survival, and response rates in patients with metastatic melanoma with BRAF V600K mutation. However, there were no cases of complete remission reported in patients with V600K mutation before.* Case Presentation*. A 53-year-old man with metastatic melanoma and dialysis dependent end stage renal failure was treated safely with Vemurafenib for a BRAF V600K mutation positive melanoma and the case was reported elsewhere. After a long follow-up of the same patient treated with Vemurafenib, a complete radiological response was observed and the renal functions remained stable throughout the treatment. Main toxicities reported were grade 1 photosensitivity and skin cancers. Vemurafenib was discontinued but patient remains disease free 12 months after stopping treatment and the clinical review is ongoing.* Conclusion*. This is the first reported case of complete radiological response to a BRAF inhibitor in metastatic melanoma with BRAF V600K mutation and remains disease free even after discontinuation of treatment. This also shows clinical safety of Vemurafenib in end stage renal failure and highlights the need for closer look at the subgroup of patients with BRAF V600K mutation and its tumour biology.

## 1. Introduction

Historically metastatic melanoma is associated with poor prognosis with a median survival of 6–10 months [1] and current treatment options mainly involve immunotherapy and targeted agents. Vemurafenib (Zelboraf) has demonstrated efficacy in treating metastatic melanoma with a known mutation in BRAF protein [2]. Approximately 40–60% of melanomas carry a BRAF mutation, which is known to enhance cell proliferation by activation of downstream signalling through MAPK pathway [3]. Around 90% of such mutation results in substitution of glutamic acid for valine at codon 600—BRAF V600E [4]. Other activating mutations are also identified such as BRAF V600K and BRAF V600D [5].

A phase 3 trial has shown improved progression-free and overall survival in previously untreated metastatic melanoma containing BRAF V600E when compared to dacarbazine chemotherapy [2]. Patients having non-V600E mutations were underrepresented in clinical trials. It has been found that patients with BRAF V600K mutation behave more aggressively and it is associated with more frequent brain and lung metastases and a shorter time from diagnosis to metastasis than other BRAF mutations [6]

Vemurafenib is highly protein bound (>99%) and is excreted via faeces (94%) and urine (1%) [7]. It has been demonstrated that Vemurafenib pharmacokinetics are not significantly altered by mild to moderate renal dysfunction. There have been no studies on Vemurafenib in patients with severe renal dysfunction except for a case report [8], as these patients were excluded from randomised clinical trials.

Here, we present an extended follow-up of a case of metastatic melanoma which was reported previously that was treated safely in end stage renal failure with Vemurafenib [8] and now we are reporting the same case after attaining complete remission.

## 2. Case Presentation

A 53-year-old male with good performance status (ECOG-0) and chronic renal failure was found to have a pigmented left parietal scalp lesion. Initial excision revealed a nodular invasive malignant melanoma with a Breslow thickness of 10 mm, 5 mitosis/mm^2^, and no lymphovascular invasion but the excision was deemed incomplete. Subsequently he underwent a wide local excision and sentinel lymph node biopsy which revealed that two out of four lymph nodes from the left supraclavicular fossa had micrometastases and so a radical neck dissection of the left neck nodes was performed. Histology showed 3 out of 29 lymph nodes positive for metastatic melanoma and mutation testing revealed presence of BRAF V600K mutation. Surgical neck wound healing was delayed due to unknown reason.

The patient was on continuous ambulatory peritoneal dialysis (CAPD) for chronic renal failure secondary to longstanding hypertension, before the diagnosis of melanoma, and his renal functions including electrolytes were stable (baseline creatinine ranged from 1004 to 1483 umol/L). There was no other significant medical history of note.

**Figure 1:**
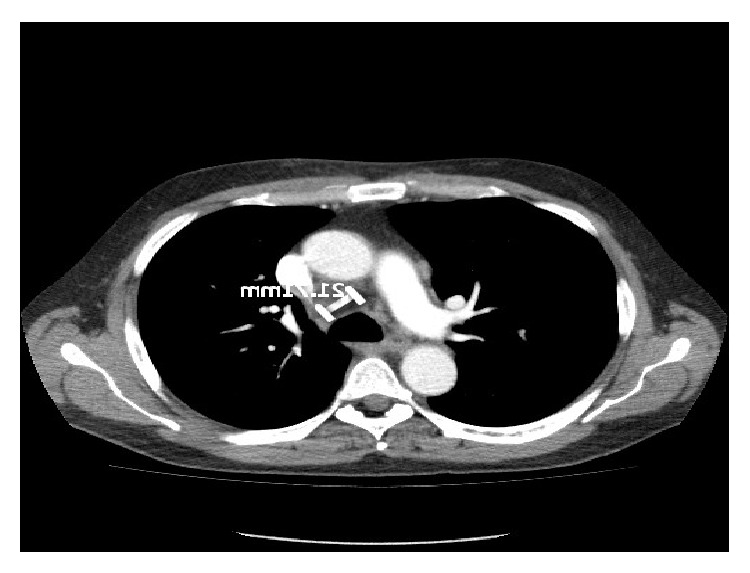
CT scan (pretreatment) showing lymph nodes.

**Figure 2:**
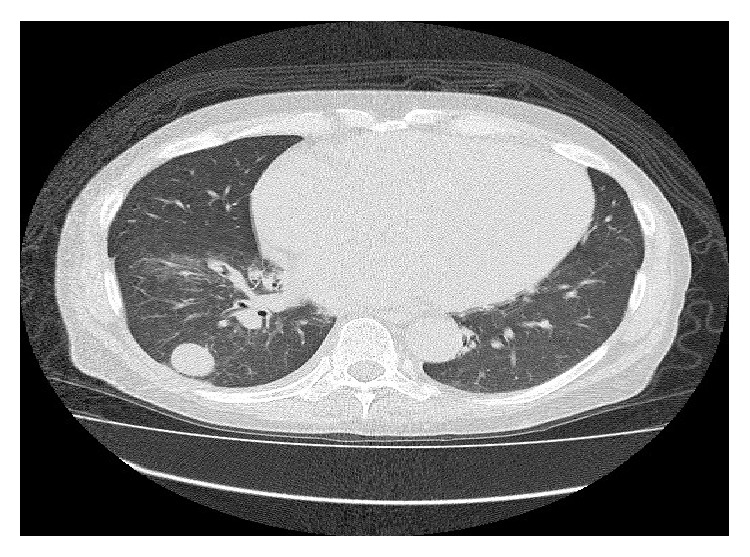
CT showing lung lesion before Vemurafenib.

Unfortunately a CT scan 3 months after his neck dissection showed convincing evidence of metastatic disease with confluent lymphadenopathy in the paratracheal group of nodes, with the target node measuring 22 mm (Figure 1). A right lower lobe lung metastasis measuring 26 mm with right perihilar lymph nodes was also noted (Figure 2). The lactate dehydrogenase (LDH) level was elevated (526 U/L) and he was then started on Vemurafenib (BRAF inhibitor) at the recommended dose (960 mg twice daily). A progress CT scan 3 months later showed significant treatment response with reduction in size of lung metastasis to 5.2 mm from 26 mm and reduction in size of mediastinal nodes. His LDH level also normalised during this period. Importantly, no significant toxicities were reported except for a grade 1 photosensitivity as the only side effect from Vemurafenib treatment.

Vemurafenib was withheld for few weeks following QTc prolongation 5 months after starting treatment but restarted at a lower dose (720 mg bd) when QTc returned to baseline. The fluctuating QTc interval was shown to be related to chronic renal failure rather than treatment related toxicity.

**Figure 3:**
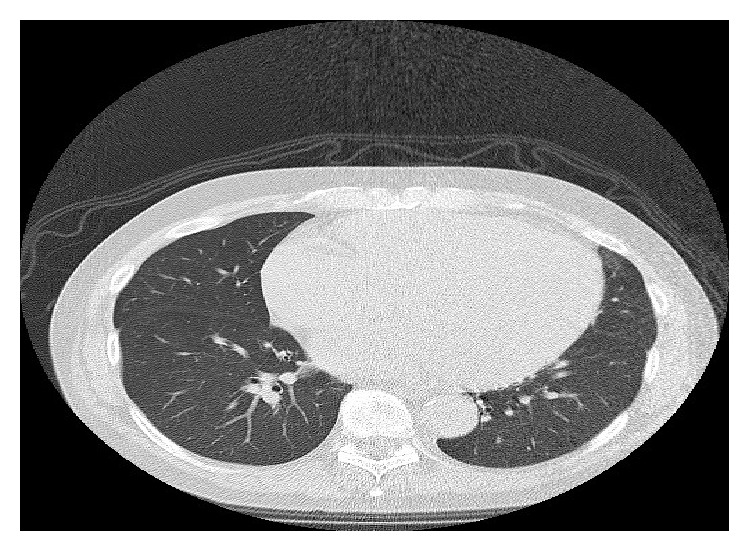
CT scan showing complete remission.

**Figure 4:**
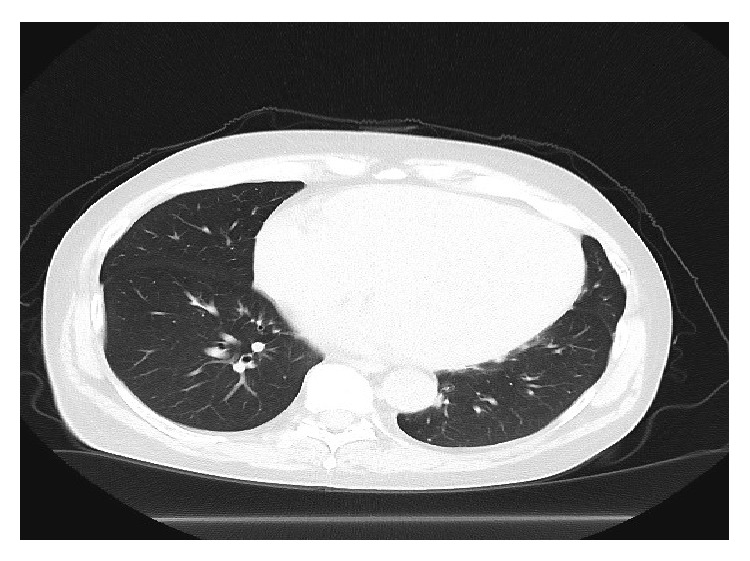
CT scan 12 months after stopping Vemurafenib.

This patient was continued on BRAF inhibitor and progress CT scans showed responding metastatic disease. A progress CT scan 2 years after starting treatment showed a complete radiological response; CT scan did not show any pathologically enlarged lymph nodes or any lung metastasis (Figure 3). The patient was also diagnosed with cardiomyopathy during Vemurafenib treatment, confirmed by echocardiogram which had shown an ejection fraction of 27%. Vemurafenib was discontinued due to cardiomyopathy and also as the patient had a complete radiological response. Most recent CT scan and clinical evaluation at 12 months after stopping Vemurafenib did not show any radiological or clinical evidence of metastatic melanoma (Figure 4).

## 3. Discussion

This is the first reported case of complete remission of metastatic melanoma on a BRAF inhibitor (Vemurafenib) in a patient with BRAF V600K mutation to the best of our knowledge. Also, this is the first report of continued complete response of metastatic melanoma with targeted therapy even after discontinuation of active therapy.

Previously, this case was reported regarding safety of Vemurafenib in end stage renal failure [8] and the same case was followed-up for 36 months and showed a sustained complete response even after stopping the BRAF inhibitor treatment. Pivotal BRIM3 trial results indicated sensitivity of Vemurafenib in BRAF V600K mutation [2]. An extended follow-up of BRIM 3 trial results had shown a median PFS of 5.9 months and median OS of 14.5 months in patients with BRAF V600K mutation. The trial also reported that 15 out of 33 patients with V600K mutation had a treatment response to Vemurafenib, but none had complete response [9].

In phase 2 trials, no patients achieved complete response to Vemurafenib in patients with BRAF V600K mutation [10]. BRAF V600K mutation carriers have poor long term prognosis when compared to V600E mutated melanoma. In all the randomised trials with BRAF inhibitors, the number of patients with BRAF V600K mutation was underrepresented to make a meaningful subgroup analysis. It is not known whether the subgroups of patients with BRAF V600K mutation behave differently to Vemurafenib treatment when compared to that of the most common BRAF V600E mutation. However, our patient had a very long progression-free survival (more than 3 yrs) with complete response and this was maintained even after stopping the BRAF inhibitor. Our patient did not show any clinical or radiological evidence of metastatic cancer 12 months after stopping Vemurafenib treatment.

This patient had also shown prolonged wound healing in the absence of a clear cause suggesting an abnormal inflammatory response likely due to immune dysregulation and the significance of which is unknown. We now know that cytokines such as IL-8 released by inflammatory cells play a crucial role in regulating cell function for host defence and also determine oncogenic properties of melanoma cells by facilitating extravasation of melanoma cells [11]. Tumour infiltrating lymphocytes (TILs) are believed to correlate with outcome, though there is debate about the applicability of this finding for all melanomas [12]. There is also limited data from randomised trials on the use of Vemurafenib in patients with severe renal impairment, although it is theoretically safe because liver is the major site of metabolism and the safety of Vemurafenib in treating melanoma patient with end stage renal failure was reported previously [8].

Our patient continues to maintain complete response after stopping Vemurafenib and this report poses the question of stopping BRAF inhibitor after complete radiological response in selected patients. This may be a cost effective way of treating metastatic melanoma with targeted agents since the patients may not require long and continuing treatment. There are no data or guidelines regarding treatment with BRAF inhibitors beyond complete response; therefore, longitudinal, controlled studies are crucial to ascertain the findings.

## 4. Conclusions

This case study shows that complete response with Vemurafenib in BRAF V600K mutation, although rare, is a possibility in selected patients and complete response was continued after stopping BRAF inhibitor treatment. This report also shows that Vemurafenib can be safely used in patients with end stage renal failure and more research is needed to address the biology of metastatic melanoma with BRAF V600K mutation.

## Abbreviations

BRAF: A gene that alters in melanoma
CTLA 4: Cytotoxic T lymphocyte associated protein 4
CT scan: Computerized tomography scan
CAPD: Continuous ambulatory peritoneal dialysis
LDH: Lactate dehydrogenase
TIL: Tumour infiltrating lymphocytes
BRIM trial: A clinical trial of Vemurafenib for treating melanoma
PFS: Progression-free survival.
